# New century: same challenges

**DOI:** 10.1038/sj.bjc.6602441

**Published:** 2005-04-05

**Authors:** F A Stillman, H Wipfli

**Affiliations:** 1 Institute for Global Tobacco Control, Johns Hopkins Bloomberg School of Public Health, 615 North Wolfe Street, Room W 6027, Baltimore, MD 21205, USA

‘If public health is to be more successful in the 21st century, it must comprehend the magnitude of the forces against it and the strategies used to engineer its defeat’ (McKinlay and Marceau, 2000b). Nowhere is this more apparent than the field of tobacco control where the transnational tobacco industry's enormous wealth and political clout are fuelling a global public health tragedy. Responsible for over 5 million premature deaths a year, tobacco-related diseases are forecasted to kill over 10 million people by 2020, 70% of these deaths occurring in low- and middle-income countries (Tobacco Free Initiative, 2004). Based on current trends, tobacco will kill 1 billion people in this century alone. Despite these sobering statistics, tobacco use continues to be promoted by a powerful industry, subsidised by governments, and seen as a normal social activity by the public.

Some may argue that the tobacco epidemic's insidiously slow onset and ‘silence’ in comparison with dramatic outbreaks of infectious diseases, such as AIDS and SARS, results in tobacco control not being given sufficient priority, prestige, or resources in public health planning and programmes. This may seem to be an explanation for why many countries have not been able or willing to take the necessary steps to control tobacco use, tobacco control capacity is inadequate, and tobacco control programmes are not adequately funded by either governments or international aid agencies. However, this is only one of many reasons. Another more important reason that explains the woefully inadequate global response to the tobacco epidemic is directly related to the aggressive activities of the transnational tobacco industry, which values corporate profits over the unimaginable damage being caused to the health and welfare of individuals and countries. The tobacco industry's past poor behaviour is well known. However, recent industry initiatives aimed at remaking its corporate image from pariah statues into a responsible corporate citizen camouflages its continued efforts to resist restrictive legislation that could lead to regulation and reduction in consumption. The industry continues to attempt to realign national and international laws so as to enlarge its corporate rights and reduce its corporate responsibilities, and undermine existing regulations.

## TOBACCO INDUSTRY INVESTMENT

Tobacco is the only legal product that, when used as intended by its manufactures, eventually kills half its users (Beaglehole and Yach, 2003). In 2004, 75% of the world's cigarette market was controlled by just four companies: Philip Morris (PM), British American Tobacco (BAT), Japan Tobacco (JT), and the China National Tobacco Corporation. The latter's share is attributed almost entirely to its near monopoly over the enormous Chinese market, but the others have been tireless in their pursuit of worldwide sales. PM, BAT, and JT have combined tobacco sales of over US$121 billion (World Bank, 2003).

It is not inconsequential that revenues generated by the multinational tobacco companies dwarf the entire gross national products (GNP) of some low- and middle-income countries. Using their tremendous economic might, transnational tobacco companies have used trade liberalisation, foreign direct investment, and marketing to take advantage of economically weak countries and expand their global market. The transnational tobacco companies have taken advantage of pressure on low- and middle-income countries from the International Monetary Fund (IMF) and World Bank to privatise state-owned industries and liberalise foreign investment laws, as well as countries’ desperate need for foreign exchange earnings. The industry penetration of formally nationalised tobacco markets has had a dramatic impact on tobacco prevalence and use. When questioned about the ethics of targeting the world's poor, a manager at Rothmans Export Ltd (part of BAT) replied: ‘It would be stupid to ignore a growing market. I can’t answer the moral dilemma. We are in the business of pleasing our shareholders’ (Sweeney, 1988).

## INDUSTRY LOBBYING

As a result of tobacco industry investment, governments view tobacco production and sales as necessary for economic development. Industry representatives also work hard to convince governments that effective control measures will result in loss of revenue from tobacco sales despite evidence that shows that tobacco control policies tend to benefit the economy rather than to cause harm (Jha and Chaloupka, 1999). The tobacco industry's size and wealth allows it to utilise many strategies and tactics to promote its interests and also to interfere with public health and tobacco control. A good deal of research has documented, at least qualitatively, the tobacco industry's specific actions to prevent or undermine tobacco control programmes and organisations. Some of these strategies and tactics include: (1) intimidation of legislative bodies through political contributions, (2) the use of lawsuit threats against whistle blowing media, (3) the use of front groups, (4) reframing public debate from health to economic and personal freedoms issues, (5) the obfuscation of science, (6) peddling of influence, (7) use of voluntary codes and pre-emptive legislation, and (8) opening markets through trade sanctions and corruption (Saloojee and Dagli, 2000; Trochim *et al*, 2003). In 2002, an expert committee convened by the World Health Organization (WHO) found that tobacco companies had slowed and undermined effective tobacco control programmes around the world and that the tobacco companies’ subversion of WHO's activities had resulted in ‘significant harm’ to global public health (World Health Organization, 2000). Despite the negative attention, the industry has not halted these type of activities and is now ramping up efforts to circumvent the World Health Organization's Framework Convention on Tobacco Control (Framework Convention on Tobacco Control, 2003).

## CIRCUMVENTING POLICY

February 2005 marks a critical achievement to stem the global tobacco epidemic, as 40 countries have ratified the WHO FCTC and the treaty will go into effect. The WHO FCTC represents the first time that low-, medium-, and high-income countries have united to develop a collective response to reduce an epidemic of chronic disease. The final WHO FCTC text commits Member States to implementing proven tobacco control measures such as increasing prices and taxes, imposing bans on advertising, promotion, and sponsorship; disclosing tobacco product components; requiring labelling standards and health warnings; promoting public education and awareness campaigns; and conducting research and surveillance programmes.

The WHO FCTC is in essence an attempt to develop a form of health governance capable of effectively regulating transnational corporations (Collin, 2002). However, there are already some indications that tobacco companies are preparing to circumvent it. In Africa, for example, we have seen the industry offer to paint buildings in the colour of specific brands to circumvent the restrictions on advertising. They are also increasingly point-of-sales displays such as ‘Power Walls’ or ‘Tobacco Product Displays’ to advertise their products. They have been challenging graphic warnings as infringements on trademark rights and are fighting to reserve public areas for smoking through their courtesy of choice campaigns and by pushing for the privatisation of public space.

## CORPORATE SOCIAL RESPONSIBILITY

Regardless of these activities, the companies are working to improve their public image through new Corporate Social Responsibly (CSR) programmes. Through these programmes, the transnational tobacco companies are acknowledging the scientific literature on the health effects of active smoking and funding youth prevention programmes in schools. They are also sponsoring the arts, and supporting community programmes. There is evidence that these programmes are working to improve their image. By 2004, the stock value of Philip Morris/Altria completely recovered from losses incurred as a result of past litigation and stockholder distrust. In 2004, BAT was awarded the Stakeholder Communication Award in the new PricewaterhouseCoopers Building Public Trust Awards (Broughton, 2004). The industry's ability to remake its image while continuing to promote aggressively a deadly product, resist new regulations and circumvent existing laws continue to be challenges for future tobacco control efforts.

## CONCLUSION – TOBACCO CONTROL BEYOND THE INDUSTRY

Despite industry efforts to undermine science and to inhibit the implementation of effective regulation, a strong evidence base regarding the health effects of active and passive smoking on human health has been established, much progress has been made in understanding which tobacco control strategies are effective and the WHO FCTC serves as a global platform on which states can develop comprehensive tobacco control programmes. However, changing a complex and addictive behavior, such as smoking, is challenging even in the absence of a global industry promoting it. New approaches and perspectives are needed. As researcher John McKinley summarises, ‘The perspectives and methods developed during the infectious and chronic disease eras have limited utility in the face of newly emerging challenges to public health’ (McKinlay and Marceau, 2000a). In regards to tobacco, we now must adopt a more systems perspective, which takes into consideration the social, cultural, economic, political, and environmental factors that influence tobacco use including the tremendous influence exerted by the transnational tobacco industry on the behaviour of individuals as well as entire countries. Such approaches to reduce tobacco use must be comprehensive and investing in building country capacity to deliver tobacco control as well as focusing efforts on developing strong policies has proven to be effective in reducing tobacco use (Stillman *et al*, 2003). There is a tremendous need to turn the knowledge base that has been established over the last decades into sustainable action. Such work will demand a renewed focus on resource mobilisation at national and international levels as well as effective cooperation, collaboration, and coordination between tobacco control stakeholders in order to maximise the scarce resources available (Stillman *et al*, in press). It is crucial that the international tobacco control community now refocus its efforts to ensure that individual countries have the knowledge, tools, data, people, and organisations needed to implement the FCTC and counter the activities of the transnational tobacco industry (Wipfli *et al*, 2004).
